# Mitigation of H_**2**_O_**2**_-Induced Mitochondrial-Mediated Apoptosis in NG108-15 Cells by Novel Mesuagenin C from *Mesua kunstleri* (King) Kosterm

**DOI:** 10.1155/2012/156521

**Published:** 2012-07-17

**Authors:** Gomathi Chan, Muhamad Noor Alfarizal Kamarudin, Daniel Zin Hua Wong, Nor Hadiani Ismail, Faizuri Abdul Latif, Aurengzeb Hasan, Khalijah Awang, Habsah Abdul Kadir

**Affiliations:** ^1^Department of Chemistry, Faculty of Science, University of Malaya, 50603 Kuala Lumpur, Malaysia; ^2^Biomolecular Research Group, Biochemistry Program, Institute of Biological Sciences, Faculty of Science, University of Malaya, 50603 Kuala Lumpur, Malaysia; ^3^Faculty of Applied Sciences, Universiti Teknologi MARA, Selangor, 40450 Shah Alam, Malaysia; ^4^Department of Aqidah and Islamic Thoughts, Academy of Islamic Studies, University of Malaya, 50603 Kuala Lumpur, Malaysia

## Abstract

This study was aimed to isolate and evaluate neuroprotective compounds from the hexane extract of the bark of *Mesua kunstleri* (Clusiaceae) on H_2_O_2_-induced apoptosis in NG108-15 cells. Five 4-phenylcoumarins were isolated by using various chromatographic techniques via neuroprotective activity-guided fractionation and isolation from the active hexane extract. The chemical structures of the isolated compounds were confirmed by NMR spectroscopic data interpretation and comparison with literature values. Cell viability data demonstrated that mesuagenin C **3** significantly increased cell viability. Hoechst 33342/PI staining illustrated mesuagenin C **3** was able to abate the nuclear shrinkage, chromatin condensation and formation of apoptotic bodies. Pretreatment with mesuagenin C **3** reduced total annexin V positive cells and increased the level of intracellular glutathione (GSH). Mesuagenin C **3** attenuated membrane potential (Δ*ψm*), reduced Bax/Bcl-2 ratio and inactivated of caspase-3/7 and -9. These results indicated that mesuagenin C **3** could protect NG108-15 cells against H_2_O_2_-induced apoptosis by increasing intracellular GSH level, aggrandizing Δ*ψm*, and modulating apoptotic signalling pathway through Bcl-2 family and caspase-3/7 and -9. These findings confirmed the involvement of intrinsic apoptotic pathway in H_2_O_2_-induced apoptosis and suggested that mesuagenin C **3** may have potential therapeutic properties for neurodegenerative diseases.

## 1. Introduction

A decade after stepping into the new millennium, neurodegenerative diseases still denote as one of world most arduous and appalling health issues as a result of increasing demographic fluctuation towards the aged population as well as growing apprehension for a better quality of life. Such alertness, together with the huge social and economic costs of the disease, has ignited in-depth research efforts to combat or at least delay the onset of neurodegenerative diseases [1]. In a normal cycle of tissue proliferation, the aged and older cells are set to decease in order to give way for the generation of new cells. Such setting of programmed cell death or better known as apoptosis was shown to be the prime cause in neurodegenerative diseases as a number of these diseases are characterized by a progressive fading away of neurons. 

Apoptosis holds a cardinal role in the maturation of the nervous system as well as neural architecture through the interplay between both anti- and proapoptotic proteins [2]. Basically, neurons either will render an adaptive response or they would initiate apoptosis when they are exposed to stress signals or apoptotic stimuli such as withdrawal of neurotrophic factor, ischemic stroke, misfolded proteins, mitochondrial-complex inhibition, excessive calcium entry, and excitotoxicity [2–4]. For example, in adult neurodegenerative disorders the formation of misfolded proteins such as *β*-amyloid aggregates brings upon oxidative stress and excessive influx of intracellular Ca^2+^ concentration which would eventually lead to apoptosis [1]. Apoptosis or programmed cell death is delineated with lucid and perspicuous morphological features, including cell shrinkage, chromatin condensation, loss of nuclear membrane integrity, plasma membrane blebbing, and eventually the breaking off of cellular fragments giving rise to the formation of apoptotic bodies [4, 5]. Naturally, apoptosis is required for normal embryonic development, sustenance of cellular homeostasis, and it has been shown to be implicated in many forms of chronic diseases. Apoptosis can take place in the body as a mean of defense mechanism in immune reactions when cells are harmed by toxic and deleterious substances [6]. Increasing substantial evidence and findings from stem cell model suggest that the fate of neural stem particularly apoptosis plays a major role in disease outcomes. Therefore, it is noteworthy that apoptosis orchestrates a crucial role in numerous neurodegenerative diseases and targeting its pathway would confer a mode of prevention and treatment [2, 4].

Generally, apoptosis is regulated by the Bcl-2 family of proteins which are further grouped into antiapoptotic proteins (Bcl-2, Mcl-1) and proapoptotic proteins (Bax, Noxa), the adaptor protein Apaf-1 (apoptotic protease-activating factor 1), and the cysteine-aspartyl-specific proteases family (caspases) [5]. Neuronal apoptosis can occur in two common pathways either by an extrinsic or intrinsic pathway and each is characterized by the alterations in the mitochondria, the endoplasmic reticulum, and cascade activation of caspases. Upon apoptotic stimuli, the proapoptotic proteins will induce the alteration in mitochondria which brings about the intensification of the outer mitochondrial membrane permeability eventuating in the generation of mitochondrial pores. The subsequent efflux of cytochrome c will bind to the Apaf-1, forming an apoptosome which triggers initiator caspase such as procaspase-9 and activating it to caspase-9. This in turn initiates a cascade of caspase activation especially the effector caspases such as caspase-3 and -7 which are accountable for proteolytic cleavage of proteins and events leading to apoptosis [3, 5, 6]. Ergo, the prevention of neuronal apoptosis confers a mode of neuroprotection in combating and delaying the onset of neurodegenerative diseases.

Nowadays, the emergence of natural products as therapeutics agents has raised a global interest among scientists in developing newer but more importantly safer drugs particularly for neurodegenerative diseases. We have undertaken a study on the hexane extract of *Mesua kunstleri* as an effort in attaining potential therapeutic compounds from the Malaysian flora [7–9]. Listed under the Clusiaceae family, *M. kunstleri* is a forest timber plant and locally known as “penaga.” The genus *Mesua* with more than 40 species is dispersed along the region of Ceylon, India, Indo-China, Thailand, Malaysia, and Queensland, centred strongly in West Malaysia [10]. Plants that fall under the genus Mesua have been used for various complementary medicine purposes such as antiallergic, rheumatism, antidiarrhoetic, and antibacterial as they are the fruitful sources of phytochemicals such as phloroglucinols, xanthones, neoflavonoids, and coumarins. These phytochemicals particularly coumarins, which are derivatives of cinnamic acid with the presence of a benzo-*α*-pyrene skeleton, have been shown to exert multifarious biological activities such as neuromodulator, antidepressant, anti-HIV-1, anti-inflammatory, antitumor, antimicrobial, antiviral, antifungal, and hepatoprotective effects [11–13]. The bark of *M. kunstleri* has been locally used to cure dyspepsis, a chronic, recurrent pain centered in the upper abdomen and renal diseases [14]. Recently, 4-phenylcoumarins including mesuagenin C **3** from the species *M. elegans* have been shown to possess acetylcholinesterase (AChE) inhibitory activity [8] and cholinesterase inhibition has been proven to be one of the effective modes of treatment for Alzheimer's diseases [15]. Withal, the medicinal use of coumarins has been intensified through the functionalization of its aromatic center that paves the way towards the development of neoteric analogs that are capable of inhibiting *β*-amyloid [16]. In mice model, plant-derived coumarins were reported to confer the ability to mitigate intracerebroventricular injection of *β*-amyloid-induced memory impairment. Osthol from *Cnidium monnieri* was shown to protect the NG108-15 by inhibiting the voltage-dependent L-type Ca^2+^ current [17] whereas isopentenyl-oxycoumarin from *Citrus *species salvaged the neuronal mixed cortical cell culture model against NMDA-induced neurotoxicity [18]. In primary cultured rat cortical model, marmesin from *Angelica gigas* was reported to act as neuroprotective agent in the glutamate-induced neurotoxicity [19]. 

This neuroprotective-activity-guided fractionation and isolation study has driven to the isolation of five 4-phenylcoumarins, namely, isomammeisin (**1**) [11], mammea A/BA (**2**) [20], mesuagenin C (**3**) [8], 5,7-dihydroxy-8-(2-methylbutanoyl)-6-[(E)-3,7-dimethylocta-2,6-dienyl]-4-phenyl-2*H*-chromen-2-one (**4**) [11], and 5,7-dihydroxy-8-(3-methylbutanoyl)-6-[(E)-3,7-dimethylocta-2,6-dienyl]-4-phenyl-2*H*-chromen-2-one (**5**) [11]. Among all these compounds, compound **3** (mesuagenin C) showed a potent neuroprotective activity against H_2_O_2_ insult, henceforth it was subjected to various assays to elucidate its neuroprotective mechanisms. To the best of our knowledge, this is the first report of neuroprotective activity in NG108-15 cells by mesuagenin C **3**. Thus, the novel neuroprotective mesuagenin C **3** could lead us to the discovery of novel molecules that potentially can act as new target sites in combating neurodegenerative diseases. 

## 2. Material and Methods

### 2.1. Plant Material

The bark of *M. kunstleri* (King) Kosterm was collected from Rimba Teloi Forest Reserve, Kedah, Malaysia on April 1995. The sample with voucher specimen number KL 4485 was identified by Mr. Teo Leong Eng and deposited in the herbarium of the Department of Chemistry, Faculty of Science, University of Malaya.

### 2.2. The Crude Extraction of *M. kunstleri* Bark

Dried ground bark of *M. kunstleri* (1.5 kg) was soaked with hexane, ethyl acetate, and methanol successively (3 × 4 L, each 48 h) at room temperature. The extracts were evaporated to dryness using a rotary evaporator. A yellow gummy residue (110.4 g) was obtained from the hexane extract. A brown gummy residue of ethyl acetate extract (70.1 g) was obtained whereas a brown amorphous powder (80.3 g)was attained from the methanol extract. 

### 2.3. Neuroprotective-Activity-Guided Fractionation and Isolation of Mesuagenin C **3**

The hexane, ethyl acetate, and methanol extracts were tested for their neuroprotective activity through MTT neuroprotective assay. Based on the results obtained, the hexane extract was selected for further fractionation since it displayed the most potent neuroprotective activity and this was carried out through neuroprotective-activity-guided fractionation and isolation. A portion of the hexane crude (10.0 g) was subjected to column chromatography fractionation over silica gel (230–400 mesh) and eluted with hexane : EtOAc (9.5 : 0.5 to 0 : 10) and EtOAc : MeOH (5 : 5) to yield fractions BH1–BH6. Fraction BH1 showed the most potent neuroprotective activity; consequently BH1 was then directed to silica gel chromatography and eluted with hexane-EtOAc (9.7 : 0.3 to 9.4 : 0.6) to produce subfractions BH1-a and BH1-b. Both of these subfractions were subjected for neuroprotective assay and the most active subfraction (BH1-b) was subjected to HPLC analysis by using ZORBAX Eclipse Plus C18, 4.6 mm i.d. × 150 mm × 3.5 *μ*m HPLC column, and for separation by using ZORBAX Eclipse Plus C18, 9.4 mm i.d. × 250 mm × 3.5 *μ*m HPLC column (3 mL/min), eluted as isocratic system with MeOH : H_2_O (both + 0.1% formic acid) 90 : 10 for 45 min to afford compounds **1**,** 2**,** 3**, **4**, and **5**. The five compounds were then subjected to MTT neuroprotective assay.

### 2.4. General Methods of Isolation and Structural Elucidation of Compounds

JEOL LA400 FT-NMR and JEOL ECA400 FT-NMR Spectrometer System (400 MHz) with CDCl_3_ as solvent were used to obtain the nuclear magnetic resonance (NMR) spectra. Mass spectra were obtained using Agilent Technologies 6530 Accurate-Mass Q-TOF Liquid Chromatography/Mass Spectrometry (LC-MS), with ZORBAX Eclipse XDB-C18 Rapid Resolution HT 4.6 mm i.d. × 50 mm × 1.8 *μ*m column. Column chromatography separations were conducted by using Merck silica gel 60 (230–400 mesh) and silica gel 60 F_254_ plates for thin layer chromatography (TLC) monitorings. In order to conduct high-performance liquid chromatography (HPLC) separation, Waters autopurification system was used and the HPLC was set up with Binary Gradient Module (Waters 2545), System Fluidics Organizer (Waters SFO), and Photodiode Array Detector (190–600 nm, Waters 2998) and Sample Manager (Waters 2767). HPLC analysis and separations were performed using ZORBAX Eclipse Plus C18 (4.6 mm i.d. × 150 mm × 3.5 *μ*m) and ZORBAX Eclipse Plus C18 (9.4 mm i.d. × 250 mm × 3.5 *μ*m) HPLC columns.

### 2.5. Cell Culture

Mouse neuroblastoma X rat glioma hybridoma cell line (NG108-15) was procured from American Type Culture Collection (ATCC). A complete medium DMEM (Dulbecco's Modified Eagle's Medium) (Sigma Aldrich) composed of 10% (v/v) heat inactivated foetal bovine serum (PAA Laboratories, Austria), 2% penicillin/streptomycin (PAA Laboratories, Austria), 1% amphotericin B (PAA Laboratories, Austria), and HAT (hypoxanthine-aminopterin-thymidine) (Sigma) was prepared and used to culture NG108-15 hybridoma cells. The complete DMEM media were filter-sterilized by using a 0.22 *μ*m filter membrane (Minisart, Sartorius Stedim). NG108-15 cells were cultured and conditioned at 5% CO_2_ moist atmosphere at 37°C (CO_2_ incubator chamber, RSBiotech) and subcultivation was conducted every 3-4 days. The cells were harvested by accutase (Innovative Cell Technologies, Inc.). The cells were daily inspected under inverted microscope (Motic) for detection of any contamination. For experimental purpose, only cells that were at their exponential growth phase (60–70% confluency) were selected. Cell viability was determined by using trypan blue with a haemocytometer. DMSO (dimethyl sulfoxide) (Sigma Aldrich) concentration in all experiments was maintained ≤0.5% v/v. 

### 2.6. MTT Assay for Assessment of Neuroprotective Activity of Mesuagenin C **3**

The neuroprotective effects of mesuagenin C **3** on the cell viability of H_2_O_2_-stressed NG108-15 cells were analyzed by MTT [3-(4,5-dimethylthiazol-2-yl)-2,5-diphenyltetrazolium bromide] assay. NG108-15 cells were raised to confluency, harvested by accutase, rinsed with PBS, and plated at a total density of 5 × 10^3^ cells/well in a 96-well plate. The cells were left to adhere for 48 h and then preincubated for 2 h with mesuagenin C **3** prior to H_2_O_2_ (2 *μ*M) exposure for subsequent 10 h. To each well 20 *μ*L MTT solution (5 mg/mL) (Sigma Aldrich) was added and incubated at 37°C for another 4 h. The cells were analyzed by using a microplate reader (ASYS UVM340) at 570 nm (with a reference wavelength of 650 nm). 

Cell viability was calculated based on the following formula:

(1) %  of  cell  viability  =[absorbance  of  treated  cells  (As)absorbance  of  control  cells  (Ac)]×100%.

### 2.7. Nuclear Double Staining with Hoechst 33342/PI

A total of 0.5 × 10^6^ cells were plated and pretreated with mesuagenin C **3** (12.5–50 *μ*M) for 2 h before exposure to H_2_O_2_ (400 *μ*M) for 10 h. After treatment, cells were harvested and washed with PBS. Hoechst 33342 (Sigma Aldrich), a DNA fluorochrome (10 *μ*g/mL), was added, followed by PI (2.5 *μ*g/mL), and the cells were further incubated for 15 min at 37°C. The cells were then observed by fluorescent microscope (Leica Inverted Fluorescence Microscope, DM16000B) and approximately 100 cells from five random microscopic fields were counted. The use of PI enabled us to differentiate the cells at different stages of apoptosis. Cells that were stained brightly by Hoechst 33342 were considered as early apoptotic cells. These cells exhibited reduced nuclear size, chromatin condensation, and nuclear fragmentation. On the contrary, cells that were stained with both Hoechst 33342 and PI were considered to be at the late apoptosis.

### 2.8. Detection of Phosphatidylserine Externalization by Annexin V-FITC/PI Staining

During apoptosis, the phosphatidylserine is translocated from the inner leaflet to the outer leaflet of the plasma membrane. Thus, annexin V-FITC/PI (BD) staining was performed to aid the detection of the apoptotic cells with translocated phosphatidylserine. Cells were stained with fluorescein-isothiocyanate- (FITC-) labelled annexin V (green fluorescence), simultaneously with dye exclusion of PI (negative for red fluorescence). Similarly, cells were plated into each 60 mm culture dishes and pretreated with mesuagenin C **3** (12.5–50 *μ*M) for 2 h prior to exposure to H_2_O_2_ (400 *μ*M) for 10 h. Cells were then harvested, gently washed, and resuspended in annexin V binding buffer (BD). Next, cells were stained with annexin V-FITC and PI in the dark at room temperature for 15 min. Cells were evaluated by using flow cytometry (BD FACScalibur). The differentiation of apoptotic and necrotic cells was based on the staining with PI. 

### 2.9. Effect of Mesuagenin**  **C **3** on Total Intracellular Glutathione (GSH) Content

Similarly, NG108-15 cells were seeded into 60 mm culture dishes and left for 48 h to adhere. The cells were then pretreated for 2 h with mesuagenin C **3** (12.5–50 *μ*M) or dimethyl sulfoxide (DMSO, 0.5%) as untreated control and were subsequently subjected to oxidative stress by exposing to 400 *μ*M H_2_O_2_ for 10 h. Cells were rinsed, harvested, and pellet was collected by centrifugation. The pellet was washed with ice-cold PBS and resuspended in 500 *μ*L of 5% 5-sulfosalicylic acid (SSA, Sigma Aldrich). The cell suspension was then centrifuged at 10 000 rpm for 15 min and supernatant was collected to be used in the intracellular glutathione assay using 96-well plate format. The supernatant was added into each well containing GSH standards (Sigma Aldrich), 5,5-dithio-bis(2-nitrobenzoic acid) (DTNB, Sigma Aldrich), and NADPH (Calbiochem) in phosphate buffer. The reaction was immediately initiated by the addition of glutathione reductase (Sigma Aldrich). The final concentrations of the reaction mixture were 95 mM potassium phosphate buffer (pH 7.0), 0.95 mM EDTA, 0.038 mg/mL (48 *μ*M) NADPH, 0.031 mg/mL DTNB, 0.115 units/mL glutathione reductase, and 0.24% 5-SSA. Absorbance was read at 1 min interval for 15 min at 405 nm with Oasys UVM340 microplate reader. The GSH concentration in each sample was calculated and compared with GSH standard curve.

### 2.10. Dissipation of Mitochondrial Membrane Potential (*Δ*ψ*m*) Analysis

In an attempt to quantify the change in mitochondrial membrane potential (*Δ*ψm**), we used JC-1 kit according to the manufacturer's protocol (Stratagene) to signal the loss of *Δ*ψm**, using the lipophylic cationic fluorescent compound JC-1 (5,5′,6,6′-tetrachloro-1,1′,3,3′-tetraethyl benzimidazolylcarbocyanine iodide). JC-1 possesses the ability to penetrate the plasma membrane and enters the cytosol of viable cell where it binds to intact mitochondrial membranes with large *Δ*ψm** to form J-aggregates which emit red fluorescence at 585 nm when excited. During membrane depolarization these J-aggregates dissociate into monomeric form which emits green color at 530 nm. Cells were harvested, washed, and stained with JC-1 for 15 min at 37°C. Cells were then washed with PBS and *Δ*ψm** was measured by flow cytometry (BD FACScalibur) for the detection of red and green fluorescence signals. JC-1 aggregates (red fluorescence) within the mitochondria of healthy cells were detected in the FL-2 channel whereas JC-1 monomers (green fluorescence) in the cytoplasm of apoptotic cells were detected in the FL-1 channel. 

### 2.11. Flow Cytometric Immunofluorescence Staining of Bax and Bcl-2 Proteins

Neuronal apoptosis is regulated by the interplay between the pro- (Bax, Bik) and antiapoptotic (Bcl-2, Bcl-xL) proteins. The ratio of these pro- and antiapoptotic proteins will decide the fate of the neurons. We next determined the protein expression of proapoptotic Bax and antiapoptotic Bcl-2 by using flow cytometric immunofluorescence staining according to the manufacturer protocol (Santa Cruz Biotechnology, Inc.). Cells were plated and pretreated with mesuagenin C **3** (12.5–50 *μ*M) for 2 h before they were exposed to H_2_O_2_ (400 *μ*M) for 10 h. After treatment, the cells were harvested, washed with ice-cold PBS, fixed, and permeabilized using intracellular flow cytometry (FCM) System (Santa Cruz Biotechnology, Inc.). Cells were resuspended and aliquoted (1.0 × 10^6^ cells/mL) into 100 *μ*L using FCM wash buffer. Next, cells were either incubated with 20 *μ*L of fluorescein-isothiocyanate- (FITC-) conjugated Bax mouse monoclonal antibody (Santa Cruz Biotechnology, Inc.) or rabbit IgG isotype control (Abcam) for 1 h. Similarly, for the expression of Bcl-2, cells were incubated with either PE-conjugated Bcl-2 mouse monoclonal antibody (Santa Cruz Biotechnology, Inc.) or rabbit IgG isotype control (Abcam) for 1 h. Cells were then washed and resuspended in 500 *μ*L FCM wash buffer. Cells were analyzed using BD Accuri C6 Flow Cytometry and BD CFlow Software.

### 2.12. Assessment of Caspase-3/7 and -9 Activities

Caspases are key mediators of cell death and caspase-3 is an executioner for apoptosis in cortical neurons in response to various insults. The dissipation of *Δ*ψm** is preceded with the leakage of cytochrome c that results in the formation of apoptosome and cascade activation of caspases. In elucidating the neuroprotective mechanism of mesuagenin C **3**, we determined caspase-3/7 and -9 activities by using Carboxyfluorescein FLICA Apoptosis Detection Kit based on the manufacturer's protocol (Immunochemistry Technologies, LLC). The enzyme activity was measured based on inhibitor probes, FAM-DEVD-FMK (FLICA) and FAM-LEHD-FMK, for caspase-3/7 and -9, respectively. These probes bind covalently and irreversibly to the active site of the active caspase heterodimer emitting the green fluorescent signal detected by the FL-1 which is a direct measure of the number of the active caspase enzymes. For this assay, the cells were pretreated with mesuagenin C **3** (12.5–50 *μ*M) for 2 h and subsequently exposed to H_2_O_2_ (400 *μ*M) for 10 h. Likewise, cells were harvested, washed with PBS, resuspended in media, and stained with 30X FLICA solutions. The cells were then incubated at 37°C under 5% CO_2_ for 1 h and washed twice with 1X FLICA washing solution. Cells were then resuspended in a 400 *μ*L washing buffer and fixed by the addition of 40 *μ*L fixative solution for flow cytometry analysis. Cells were analyzed using BD Accuri C6 Flow Cytometry and BD CFlow Software.

### 2.13. Statistical Analysis

All the experimental data are expressed in mean ± standard error (S.E.). Statistical differences between groups were analyzed and calculated by one-way analysis of variance (ANOVA) from at least three independent experiments and this is followed by Dunnett's test. *P* < 0.05 was considered to be significantly different from the H_2_O_2_-treated groups.

## 3. Results

### 3.1. Hexane Fraction of *M. kunstleri* Exhibited the Strongest Neuroprotective Effect

**Figure 1:**
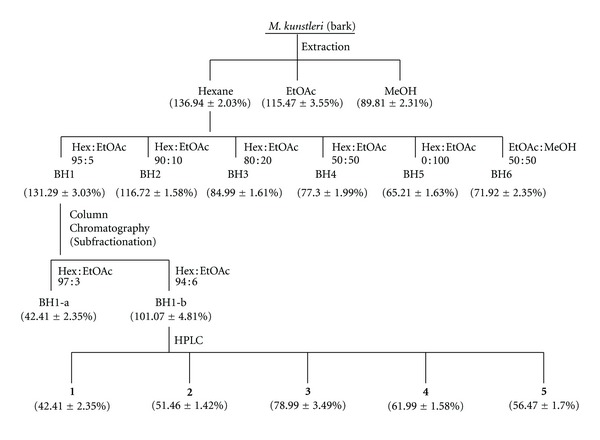
Fractionation and isolation of the bioactive compounds from the bark of *M. kunstleri*.

**Figure 2:**
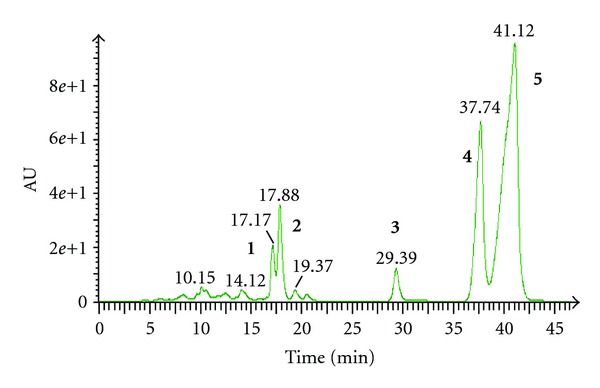
Chromatogram of BH1-b. **1.** Isomammeisin **2.** Mammea A/BA **3.** Mesuagenin C **4. **5,7-Dihydroxy-8-(2-methylbutanoyl)-6-[(E)-3,7-dimethylocta-2,6-dienyl]-4-phenyl-2*H*-chromen-2-one **5.** 5,7-Dihydroxy-8-(3-methylbutanoyl)-6-[(E)-3,7-dimethylocta-2,6-dienyl]-4-phenyl-2*H*-chromen-2-one.

**Figure 3:**
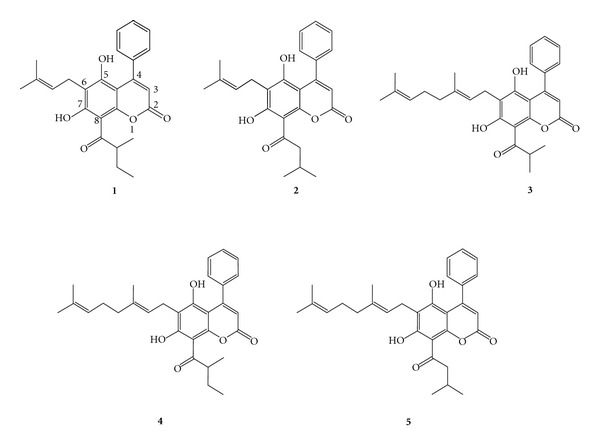
The chemical structures of the compounds isolated from *M. kunstleri* via neuroprotective activity-guided approach.

We studied the concentration-dependent effect of H_2_O_2_ challenge on cell viability for 10 h by using MTT cell viability assay. NG108-15 cells were treated with different concentrations of H_2_O_2_ (0.1–4 mM) for 10 h and a significant dose-dependent reduction in cell viability was detected. After exposure to 2 mM H_2_O_2_, NG108-15 cell viability was about 45.41 ± 3.42% of the control viability. Pretreatment (2 h) with BH extract showed the highest percentage cell viability of 136.94 ± 2.03%. The BH extract was then subjected to neuroprotective activity-guided fractionation and isolation approach due to its highest neuroprotective-activity and six fractions were obtained after fractionation (Figure 1). Fraction BH1 possessed the strongest neuroprotective activity among the fractions with the percentage cell viability of 131.29 ± 3.03%. Fractionation of BH1 yielded two subfractions (BH1-a and BH1-b) with sub-fraction BH1-b possessing stronger neuroprotective effect (101.07 ± 4.81%) as compared with fraction BH1-a. Hence, BH1-b was further chromatographed and five pure compounds were isolated and identified successfully (Figures 2 and 3).

### 3.2. Isolation of Mesuagenin C **3** and Its Protective Effects against H_2_O_2_-Induced Apoptosis in NG108-15 Cells

Among the five compounds obtained from the fractionation of BH1-b, mesuagenin C **3** was isolated from *M. kunstleri* for the first time and its structure was confirmed by comparison of the obtained spectral data with the published literature data [8]. Results were summarized in Figures 1–3. The structure of mesuagenin C **3** was further confirmed by HRESI-MS, in a positive mode, which revealed the molecular ion peak at *m*/*z* 483.1183 [M + Na]^+^ which corresponds to a molecular formula of C_29_H_32_O_5_. The comparison of ^1^H and ^13^C NMR of mesuagenin C from literature values [8] and compound **3** was depicted in Table 1. In view of the mentioned data and comparison to the literature values, the identity of compound **3** was confirmed as mesuagenin C.

**Figure 4:**
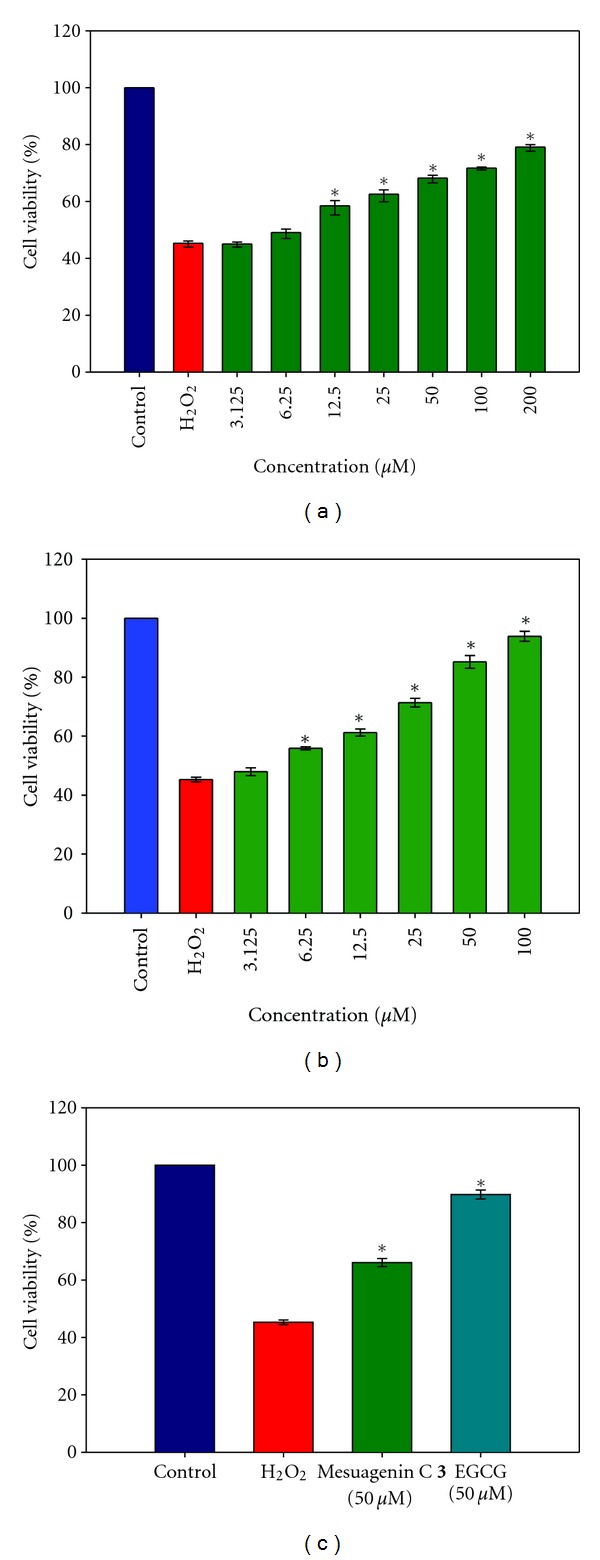
Neuroprotective effect of mesuagenin C **3** by MTT cell viability assay. (a) Dose-dependent increase in cell viability by pretreatment with mesuagenin C **3** in H_2_O_2_-induced cell death prior to 2 mM of H_2_O_2_ exposure for 10 h. (b) Dose-dependent increase in cell viability by pretreatment with EGCG prior to 2 mM of H_2_O_2_ exposure for 10 h. (c) Comparison of mesuagenin C **3** and EGCG pretreated cell viability when exposed to H_2_O_2_. NG108-15 cells were incubated for 48 h followed by pretreatment with mesuagenin C **3** (50 *μ*M) for 2 h prior to exposure to 2 mM H_2_O_2_ (10 h). EGCG (50 *μ*M) was used as a standard reference. Values are mean ± S.E. from at least three independent experiments. The asterisk indicated significantly different values from H_2_O_2_-treated cells (*P < 0.05).

Next, after the identification of the compounds, NG108-15 cells were pretreated with all the compounds at varying concentrations (3.125–200 *μ*M) for 2 h prior to exposure to of H_2_O_2_ (2 mM). We found that mesuagenin C **3** significantly increased the cell viability, up to 78.99 ± 3.49% which was the greatest neuroprotection as compared to the rest of the compounds (Table 2). Furthermore, mesuagenin C **3** dose-dependently increased cell viability as shown in Figure 4(a). Taken together, these results allow us to conclude that mesuagenin C **3** was effective for the protection and viability of NG108-15 cells. In the scope of neuroscience, the neuroprotective properties of epigallocatechin gallate (EGCG) have been demonstrated in regards to its ability to regulate cell survival and signal transduction in various models of neuroprotection [21–24]. Thus, EGCG was selected as a standard reference in the neuroprotective evaluation of all the five 4-phenylcoumarins (Figure 4(b)). Mesuagenin C **3** showed lower neuroprotective activity when compared to EGCG at 50 *μ*M (Figure 4(c)); however, the neuroprotective activity of mesuagenin C **3** at concentrations of 25 *μ*M and above was significantly different (*P* < 0.05) compared to H_2_O_2_-treated group.

### 3.3. Mesuagenin C **3** Suppressed H_2_O_2_-Induced Nuclear Morphologic Changes in NG108-15 Cells

**Figure 5:**
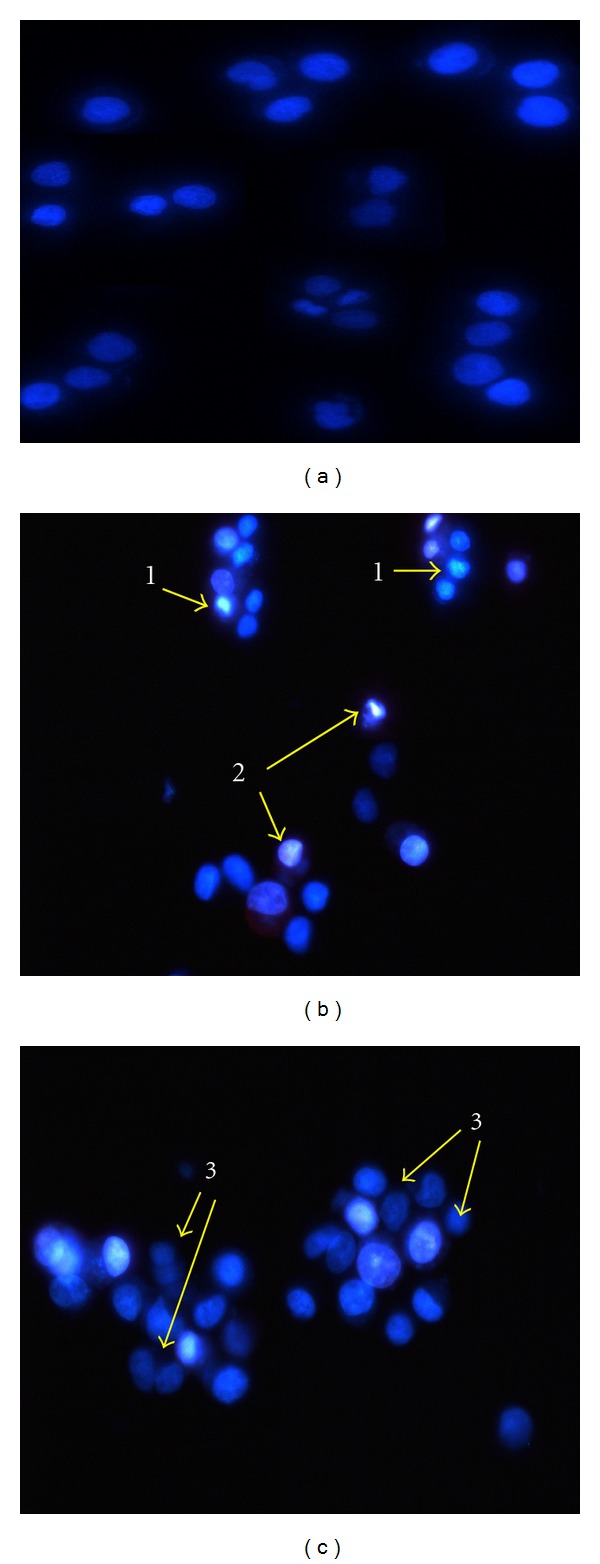
Mesuagenin C **3** prevented H_2_O_2_-induced morphological changes in NG108-15 cells. (a) Viable cell nuclei were evenly stained. (b) H_2_O_2_-treated cells show reduced nuclear size, chromatin condensation, and the nuclei were unevenly stained with intense blue fluorescence (arrow 1) which indicates early apoptotic cells. Cell nuclei that were dual stained with Hoechst 33342 and PI (arrow 2) were considered to be at their late apoptosis. (c) However, after pretreatment with mesuagenin C **3** (50 *μ*M), there was a clear reduction of apoptotic cell number (arrow 1) and increasing number of evenly stained nuclei indicating viable cells (arrow 3). This clearly proved that pretreatment with mesuagenin C **3** prevents the induction of neuronal apoptosis in NG108-15 cells (magnification 400x).

The neuroprotective effect of mesuagenin C **3** was validated through fluorescence imaging morphological analysis. To determine whether the reduced cell viability was due to apoptosis, NG108-15 cells were stained with Hoechst 33342/PI. As depicted in Figure 5(a), control (untreated) cells without H_2_O_2_ treatment were uniformly stained with and displayed equally disseminated chromatin, normal organelle, and intact cell membrane. In contrast, cells that were treated with 400 *μ*M H_2_O_2_ for 10 h (Figure 5(b)) illustrated archetypal characteristics of apoptotic cells including the condensation of chromatin, shrinkage of nuclei, and presence of apoptotic bodies with intense blue fluorescence. However, pretreatment (2 h) with varying concentrations of mesuagenin C **3** (12.5–50 *μ*M) markedly reduced the level of H_2_O_2_-induced nuclei morphological alterations, and the number of cells with nuclear condensation and fragmentation was significantly decreased (Figure 5(c)).

### 3.4. Mesuagenin C **3** Mitigated the Externalization of Phosphatidylserine

**Figure 6:**
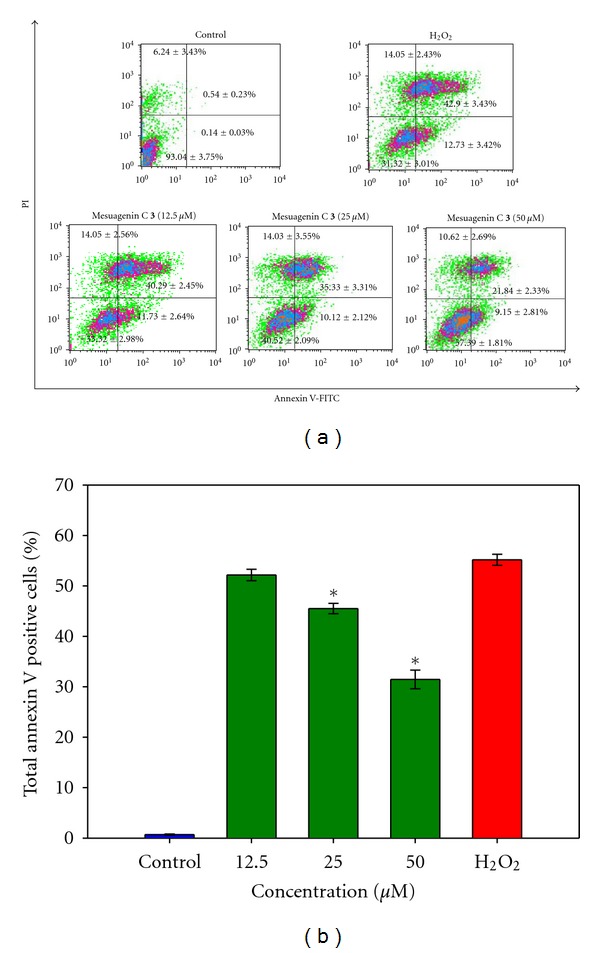
The effect of mesuagenin C **3** on the externalization of PS in NG108-15. (a) The different distribution of NG108-15 cells stained with annexin V-FITC/PI in a dual parametric dot plots of PI fluorescence (*Y*-axis) versus annexin V-FITC fluorescence (*X*-axis). (b) Bar chart indicates the proportion of annexin V positive cells (annexin V^+^/PI^−^ and annexin V^+^/PI^+^) as compared to H_2_O_2_-treated cells. Values are mean ± S.E. from at least three independent experiments. The asterisk indicated significantly different values from H_2_O_2_-treated cells (*P < 0.05).

The addition of H_2_O_2_ (400 *μ*M) significantly increased both annexin V^+^/PI^−^ (early apoptosis, lower right quadrant) and annexin V^+^/PI^+^ (late apoptosis, upper right quadrant) cell populations to 11.73 ± 3.42% and 42.90 ± 2.78%, respectively, as indicated in Figure 6(a). In contrast, pretreatment with mesuagenin C **3** (50 *μ*M) for 2 h followed by H_2_O_2_ exposure decreased the early apoptotic cell population to 9.15 ± 1.65% and late apoptotic cell population to 21.84 ± 2.33% (Figure 6(a)). Pretreatment with 12.5, 25, and 50 *μ*M of mesuagenin C **3** for 2 h followed with H_2_O_2_ insult dose-dependently reduced the cumulative early and late apoptotic cell population from 54.63 ± 2.67% to 51.99 ± 2.55%, 45.45 ± 2.72%, and 30.99 ± 3.09%, respectively, as shown in Figure 6(b). It was also noted that the pretreatment increased the viable cell population (lower left quadrant) from 38.30 ± 3.01% (H_2_O_2_-treated cells) to 57.39 ± 1.81% (Figure 6(a)). Based on these results, mesuagenin C **3** (*P < 0.05) prevented the effects of H_2_O_2_-induced apoptosis, signifying its potential neuroprotective ability in the present model.

### 3.5. Mesuagenin C **3** Dose-Dependently Aggrandized Intracellular GSH Concentration in H_2_O_2_-Treated NG108-15 Cells

**Figure 7:**
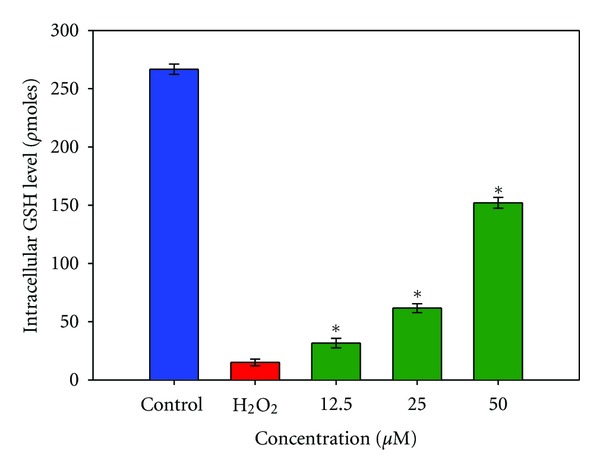
The effect of mesuagenin C **3** on total intracellular GSH level. Pretreatment with mesuagenin C **3** dose-dependently aggrandized intracellular GSH level after H_2_O_2_ challenge in the NG108-15 cells. Values are means ± S.E. from at least three independent experiments. The asterisk indicated significantly different values from H_2_O_2_-treated cells (*P < 0.05).

Since the level of intracellular GSH plays a pivotal role in the neuroprotection, we also determined the effect of mesuagenin C **3** on the intracellular GSH content in the NG108-15 cells challenged with H_2_O_2_. The intracellular GSH level for each concentration of mesuagenin C **3** (12.25–50 *μ*M) was determined from a standard curve constructed using known amounts of GSH (0.5–0.00195 nmoles). Exposure of NG108-15 cells with H_2_O_2_ significantly decreased the GSH level to 10 *ρ*moles as compared to 269 *ρ*moles in the untreated cells. In agreement with the protective effect of mesuagenin C **3**, a significant increase in the intracellular GSH level in mesuagenin C **3-**treated cells was observed. Pretreatment with mesuagenin C **3** (12.25–50 *μ*M) increased the level of intracellular GSH dose-dependently, yielding a 16.2-fold increase at the highest concentration of 50 *μ*M, as compared to the H_2_O_2_-treated cells (Figure 7).

### 3.6. Mesuagenin C **3** Attenuated H_2_O_2_-Induced Dissipation of *Δ*ψ*m*

**Figure 8:**
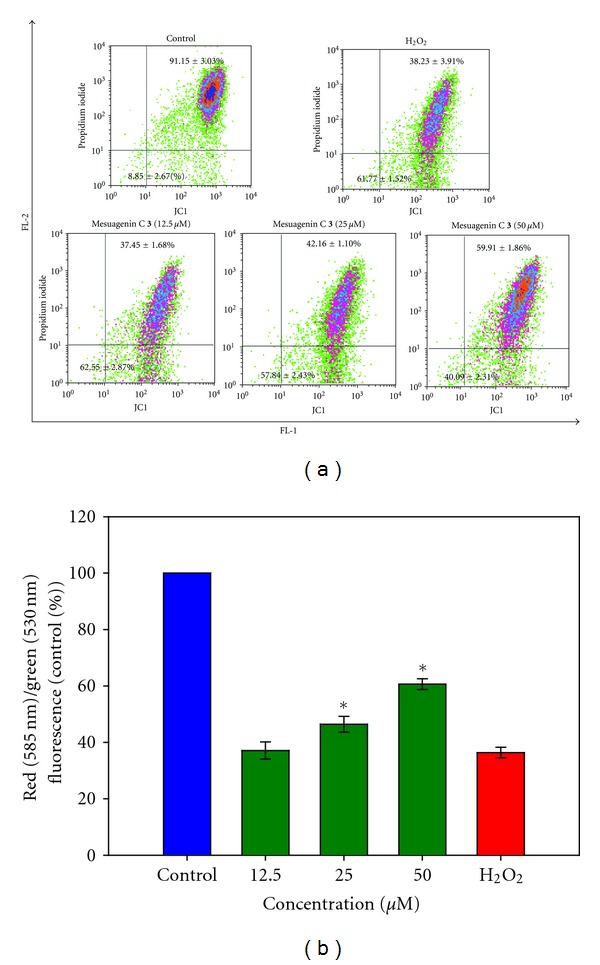
Flow cytometry analysis showing alterations in the *Δ*ψm** of NG108-15 cells. Upon completion of treatment, the cells were stained with JC-1 and the alteration in *Δ*ψm** was analyzed as mentioned in methods. (a) Representative dot plots of JC-1 aggregates (FL-2 Red fluorescence) versus JC-1 monomers (FL-1 green fluorescence). (b) Bar chart showing the percentages of relative fluorescence intensity of *Δ*ψm** in control, H_2_O_2_-only and varying pretreatment concentrations of mesuagenin C **3** in NG108-15 cells. Values are mean ± S.E. from at least three independent experiments. The asterisk indicated significantly different values from H_2_O_2_-treated cells (*P < 0.05).

The dissipation of Δ*ψm* is an apoptotic hallmark in the initiation of apoptosis, exemplifies one of the early events occurring during apoptosis. A cationic lipophilic fluorescent probe known as JC-1 was used to indicate the loss of Δ*ψm*. This lipophilic dye enters mitochondria in proportion to the membrane potential. JC-1 will form J-aggregates at the high intramitochondrial concentrations induced by higher Δ*ψm* values. From the data attained, there was an alteration of fluorescence signal from the upper right quadrant to the lower right quadrant which leads to a lower red fluorescence signal (38.23 ± 3.91%) and higher green fluorescence signal (61.77 ± 1.52%) in H_2_O_2_-treated cells, signifying disruption of *Δ*ψm** (Figure 8(a)). Nevertheless, this alteration was reversed by 2 h pretreatment with mesuagenin C **3** in a dose-dependent manner as shown in Figure 8(b) where NG108-15 cells treated with 50 *μ*M mesuagenin C **3** (*P < 0.05) overturned the effect of H_2_O_2_, shifting the fluorescence signal from lower right (40.09 ± 2.31%) to the upper right (59.91 ± 1.86%) as indicated by JC-1 fluorescence ratio bar chart (Figure 8(a)).

### 3.7. Mesuagenin C **3** Modulated the Expression of Bax and Bcl-2 Proteins in *H*_2_ *O*_2_-Stressed Cells

**Figure 9:**
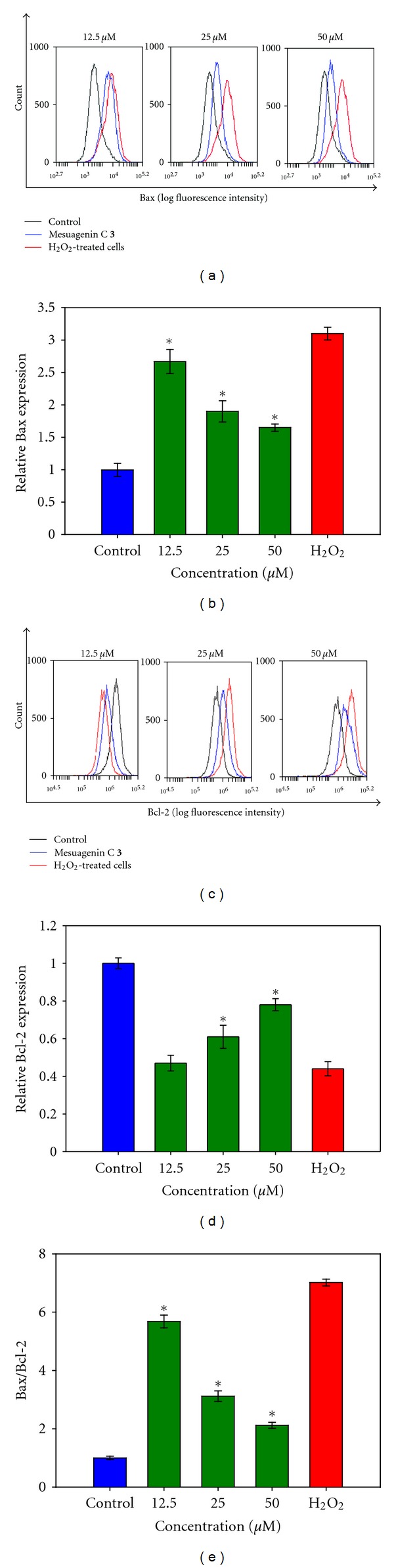
Mesuagenin C **3** modulated Bax and Bcl-2 protein expression in H_2_O_2_-treated NG108-15 cells. (a) Representative overlay of histograms showing Bax-associated immunofluorescence. (b) Bar chart represents dose-dependent downregulation of Bax protein expression after pretreatment with mesuagenin C **3**. (c) Representative overlay of histograms showing Bcl-2-associated immunofluorescence. (d) Bar chart represents dose-dependent elevation of Bcl-2 expression after pretreatment with mesuagenin C **3**. (e) Bar chart represents dose-dependent attenuation of Bax/Bcl-2 ratio. Values are mean ± S.E. from at least three independent experiments. The asterisk indicated significantly different values from H_2_O_2_-treated cells (*P < 0.05).

The ratio of proapoptotic Bax to antiapoptotic Bcl-2 is proven to be associated to the initiation of a cascade leading to the activation of caspases, such as caspase-3 which triggers apoptosis. To investigate the effects of mesuagenin C **3** on Bax/Bcl-2 ratio, NG108-15 cells were first pretreated with different concentrations of mesuagenin C **3** (12.5, 25, and 50 *μ*M) for 2 h prior to exposure to H_2_O_2_ for 10 h. Our obtained data showed that H_2_O_2_-treated cell profiles shifted to the right in the Bax histograms resulting in 3.09-fold increase (Figures 9(a) and 9(b)) in the expression of Bax protein, while decreasing the level of Bcl-2 (Figures 9(c) and 9(d)). This has resulted in a significant surge in the Bax/Bcl-2 ratio to 7.02-fold (Figure 9(e)). However, pretreatment with mesuagenin C **3** (12.5, 25 and 50 *μ*M) inhibited the H_2_O_2_-induced increase of Bax (Figures 9(a) and 9(b)) and decrease of Bcl-2 protein expression (Figures 9(c) and 9(d)) dramatically when compared to H_2_O_2_-treated cells. This is further demonstrated by the lowering in the ratio of Bax/Bcl-2 from 5.68 to 2.12 (Figure 9(e)). On that account, mesuagenin C **3** may prevent the NG108-15 cells from entering neuronal apoptosis.

### 3.8. Mesuagenin C **3** Attenuated H_2_O_2_-Induced Activation of Caspase-3/7 and -9 in H_2_O_2_-Treated NG108-15 Cells

**Figure 10:**
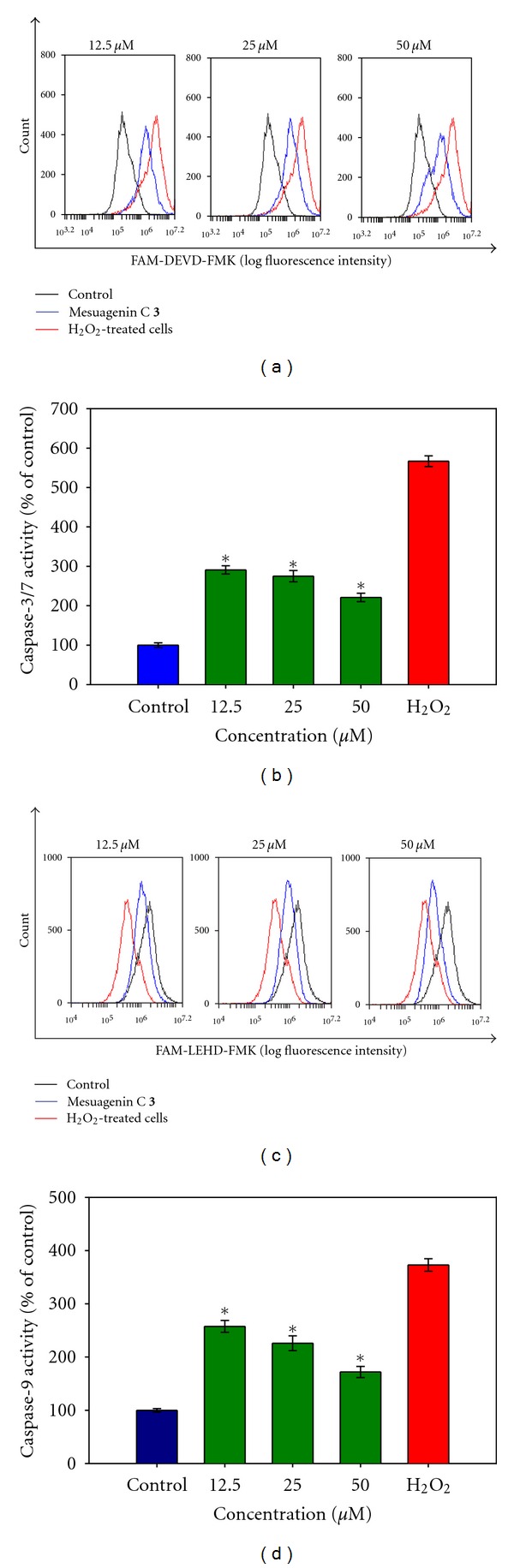
Mesuagenin C **3** prevented H_2_O_2_-induced activation of caspase-3 and caspase-9 in H_2_O_2_-treated NG108-15 cells. (a) Representative overlay of histograms showing caspase-3/7-associated immunofluorescence. (b) Bar chart represents dose-dependent reduction in caspase 3/7 activity after pretreatment with mesuagenin C **3**. (c) Representative overlay of histograms showing caspase-9-associated immunofluorescence. (d) Bar chart represents dose-dependent reduction in caspase-9 activity after pretreatment with mesuagenin C **3**. Values are mean ± S.E. from at least three independent experiments. The asterisk indicated significantly different values from H_2_O_2_-treated cells (*P < 0.05).

To investigate the involvement of caspase-dependent and caspase-independent pathways in H_2_O_2_-induced apoptosis, the NG108-15 cells were subjected to H_2_O_2_ treatment (400 *μ*M) for 10 h and the caspase-3/7 and -9 activities were measured using flow cytometry analysis. The results in Figures 10(a) and 10(c) showed that H_2_O_2_-treated cell profiles shifted to the right in caspase-3/7 and -9 histograms indicating significant aggrandization of caspase-3/7 (566.76 ± 13.59%) and caspase-9 (372.97 ± 11.62%) activities when compared to untreated control cells. We next investigated the effects of mesuagenin C **3** on elevated caspase-3/7 and -9 activities induced by H_2_O_2_. The findings demonstrated that pretreatment with mesuagenin C **3** (50 *μ*M) for 2 h partially inhibited the elevated activities of caspase-3/7 and -9 induced by H_2_O_2_. The caspase-3/7 and -9 activities were found to be significantly reduced to 221.59 ± 10.66% (Figure 10(b)) and 171.39 ± 10.56% (Figure 10(d)), respectively, (*P < 0.05), as compared to the H_2_O_2_-treated cells. The reduction of caspase-9 activity further explains the attenuation of the caspase-3/7 activation which reversed the H_2_O_2_-induced apoptosis effects. The data suggested that mesuagenin C **3** may suppress caspase-9 activation followed by inhibition of caspase-3/7 via protecting mitochondrial membrane integrity and suppressing cytochrome c release from the mitochondrial intermembrane space. The results implied that H_2_O_2_-induced apoptosis was mediated by caspase-dependent intrinsic apoptotic pathways.

## 4. Discussion 

In the present study, we found that the hexane fraction of *M. kunstleri *prevented H_2_O_2_-induced neurotoxicity in NG108-15 cells. In order to verify the neuroprotective components of *M. kunstleri*, as part of a continuing study on neuroprotection-effects of *M. kunstleri*, neuroprotective-activity-guided fractionation and isolation was carried out to search for the active fractions and compounds. We compared the various extracts on neuroprotective activity and the hexane extract of *M. kunstleri *exerted the most potent neuroprotective activity against H_2_O_2_-induced neurotoxicity in NG108-15 cells. A neuroprotective-activity-guided fractionation and isolation of the active chemical constituents led to the identification of the five components: isomammeisin (**1**), mammea A/BA (**2**), mesuagenin C (**3**), 5,7-dihydroxy-8-(2-methylbutanoyl)-6-[(E)-3,7-dimethylocta-2,6-dienyl]-4-phenyl-2H-chromen-2-one (**4**), and 5,7-dihydroxy-8-(3-methylbutanoyl)-6-[(E)-3,7-dimethylocta-2,6-dienyl]-4-phenyl-2H-chromen-2-one (**5**), in which compound **3** showed the most potent neuroprotective activity. All of the five compounds isolated in fraction F1-b possess a 4-phenylcoumarin skeleton and they differ in the substituents attached to the position C-6 and C-8. These results suggest that the C-6 side chain of the 4-phenylcoumarin is essential for the neuroprotective activity; compounds possessing free geranyl chain at position C-6 (**3**, **4**, and **5**) are active whereas compounds possessing free prenyl chain at the same position (**1** and **2**) are inactive. Based on the MTT assay, mesuagenin C **3** illustrated the most significant neuroprotective activity towards H_2_O_2_-induced cell death in the NG108-15 cells. In the present study, we focused on the effect of mesuagenin C **3** on H_2_O_2_-induced apoptosis in NG108-15 cells and attempted to elucidate the potential mechanisms underlying its neuroprotective effect.

Cells can die by two major mechanisms: necrosis or apoptosis. Apoptosis is a gene-regulated phenomenon with the characteristic alterations of cellular structure including chromatin condensation, cell and nuclear shrinkage, oligonucleosomal DNA fragmentation, and membrane blebbing [25]. The neuroprotective effect of mesuagenin C **3** was first verified using morphological analysis (Figure 5). Hoechst 33342/PI double staining was used to identify the morphological changes in apoptotic nuclei. Morphological changes associated with apoptotic cell death induced by H_2_O_2_ were characterized by the presence of shrunken cells, nuclear shrinkage, chromosome condensation, and appearance of apoptotic bodies. On the contrary, these cellular events were evidently abrogated when the cells were pretreated with mesuagenin C **3**. Externalization of phosphatidylserine is a hallmark of the changes in the cell surface during apoptosis [26]. Flow cytometric annexin V-FITC/PI double staining revealed that NG108-15 cell death after the exposure to 400 *μ*M H_2_O_2_ for 10 h was mainly through apoptosis. Having established that mesuagenin C **3** can exert its protective effect against H_2_O_2_-induced neurotoxicity in NG108-15, we studied the potential pathways involved. 

The major cellular antioxidant glutathione (GSH) holds a cardinal role in the line of defense against oxidative stress and its deficiency can sensitize the brain to injury [27–29]. Glutathione is the most luxuriant antioxidant in the brain which predominantly functions to detoxify H_2_O_2_ by decomposing it into water and oxygen and keeps the thiol groups of proteins in the reduced state [30]. Oxidative stress that may result as a repercussion of elevated intracellular levels of reactive oxygen species (ROS), such as hydrogen peroxide (H_2_O_2_), evidently forms a common pathway leading to neuronal death [31]. Numerous studies have demonstrated decreased levels of glutathione in pathological conditions including brain ischemia and neurodegenerative diseases. The balance between generation of ROS and antioxidative processes can become perturbed as reported in aging [32] and several neurological disorders such as Parkinson's disease (PD) and Alzheimer's disease (AD) [33]. A substantial growing line of evidence indicated that a high concentration of intracellular glutathione protects cells against different ROS [34]. Withal, direct depletion of ROS by glutathione, a number of associated enzymes confer varied roles for glutathione in living cells. For instance, glutathione peroxidase, glutathione reductase, transferase, and glutaredoxin all utilize glutathione in reactions that remove peroxide, as well as potential toxin, control the redox homeostasis of the cell, and regulate protein function through thiolation and dethiolation. This network of enzymes using glutathione as substrate has been implicated in DNA synthesis and repair, protein synthesis, amino acid transport, and enzyme activation or inactivation [35]. Glutathione depletion on the other hand has been shown to directly modulate both the dissipation of mitochondrial membrane potential and the activation of executioner caspases such as caspase-3/7 [36]. In this study, we demonstrated that pretreatment of the cells with mesuagenin C **3** effectively averted cell death induced by H_2_O_2_ which depleted intracellular glutathione in NG108-15 cells (Figure 7). A significant dose-dependent depletion in GSH levels was observed to be associated with apoptotic changes when the NG108-15 cells were treated with 400 *μ*M H_2_O_2_. Pretreatment with mesuagenin C **3** at 50 *μ*M significantly aggrandized the level of intracellular GSH by about 16-fold which may explain one of its mechanisms in salvaging the NG108-15 cells from severe oxidative stress damage. Concomitantly, results in this study have also established the ability of mesuagenin C **3** to ameliorate the mitochondrial membrane potential after consequential loss of potential due to H_2_O_2_ challenge. When coupled with its potential in preserving the integrity of the mitochondria membrane and through this mesuagenin C **3** directly suppresses the pore or channel formation in the outer mitochondrial membrane and thus represses the cytochrome c release leading to the disruption of the apoptotic cascade involving the Bcl-2 proteins and hence the caspases. As a result, this disrupts the downstream pathway leading to the formation of apoptosomes, hence the execution of apoptosis. 

Many current researches have been focusing on the analysis of apoptotic factors centered in mitochondria and the nucleus. The induction of apoptosis is presumably related to oxidative-stress-mediated mitochondrial dysfunction. Decreased *Δ*ψm** and reduced ATP production are linked to mitochondrial-dependent apoptotic pathway and define the mitochondrial dysfunction [37]. Oxidative injury via H_2_O_2_ has been reported to dissipate *Δ*ψm**, resulting in the rapid release of caspase activators such as cytochrome c into the cytoplasm, thus eliciting the apoptotic process [38, 39]. Interestingly, results from our study indicated that pretreatment with mesuagenin C **3 **(50 *μ*M) prior to H_2_O_2_ (400 *μ*M) effectively prevented the loss of *Δ*ψm** (Figures 8(a) and 8(b)) by decreasing the mitochondrial depolarization. These results suggest that mesuagenin C **3** may play critical roles in preventing H_2_O_2_-induced apoptosis through its protective action on the mitochondria. 

The Bcl-2 family proteins have emerged as the critical regulators of the mitochondrial-mediated apoptosis by functioning either as promoters or inhibitors of the cell death process [40]. Indeed, the two main members of Bcl-2 family, Bax and Bcl-2, have been well documented to play a vital role in the mitochondrial pathway of apoptosis. Bax has been associated in stimulating cell apoptosis, whereas Bcl-2 in inhibiting apoptosis [41, 42]. The balance between Bcl-2 and Bax expression plays an imperative role in sustaining cell morphology and function. It was reported that Bcl-2 overexpression can disturb the regulation of the proapoptotic protein, Bax [43, 44]. Bax regulates cytochrome c release from mitochondria through the formation of mitochondrial transition pore [45]. Accordingly, there is evidence advocating that Bcl-2 maintained the mitochondrial integrity, while Bax destroyed the mitochondrial integrity and caused loss of mitochondrial membrane potential [3, 4, 43], which in turn triggered neuronal programmed cell death. Furthermore, a rise in Bcl-2 expression averts cytochrome c release from the mitochondria, in so doing inhibiting activation of caspases, such as caspase-9 and caspase-3, and preventing apoptosis [44]. Therefore, cells are healthy and active when Bcl-2 is overexpressed and instead they decease when Bax is hyperexpressed [46]. As a result, Bax/Bcl-2 ratio determines the vulnerability to apoptosis and, hence, decides the fate of life and death of a cell [47]. In view of that and since mesuagenin C **3** has been proven to interfere in the loss of *Δ*ψm** (Figures 8(a) and 8(b)), we investigated mitochondrial-mediated neuroprotective mechanism of mesuagenin C **3** through the expression of Bax and Bcl-2 proteins. 

Our results show that H_2_O_2_ treatment significantly increased the expression of proapoptotic Bax and reduced the expression of antiapoptotic Bcl-2 in a dose-dependent manner, resulting in a significant increase in Bax/Bcl-2 ratio (Figures 9(a) and 9(c)). However, 2 h pretreatment with mesuagenin C **3** reversed the effects of H_2_O_2_-treated cells through the downregulation of Bax and upregulation of Bcl-2, leading to a reduction in Bax/Bcl-2 ratio (Figure 9(e)), suggesting that mesuagenin C **3** shifted the balance between pro- and antiapoptotic members toward cell survival. These results suggested that mesuagenin C **3** protects NG108-15 cells significantly via the downregulation of Bax and upregulation of Bcl-2 against H_2_O_2_-induced apoptosis in the present neuroprotection model.

An upsurge in the levels of proapoptotic proteins and/or a diminution in *Δ*ψm** and an opening of mitochondrial permeability transition pores subsequently lead to cytochrome c release from mitochondria into cytosol [48, 49]. Once released from mitochondria, cytochrome c binds to the apoptosis inducing factor and activates caspase-9 [50]. Caspase-9 is a well-known initiator caspase which is activated during apoptosis and linked to the mitochondrial-mediated death pathway [51]. After treatment of cells with apoptotic agents including H_2_O_2_, cytochrome c will be released from mitochondrial intermembrane space and binds to the apoptosis protease activation factor (APAf-1) forming an apoptosome complex. This complex activates caspase-9. Once activated, caspase-9 will then cleave procaspase-3 and procaspase-7, which are responsible for several cellular apoptosis processes [52]. Caspase-3 plays a central role in apoptosis, chromatin condensation, and DNA fragmentation [53]. Moreover, the activation of caspase-3 by H_2_O_2_ treatment *in vitro* was reported to be a crucial effector of apoptosis event [54]. In agreement with that study, the present results indicate that H_2_O_2_-induced caspase-3 activation in NG108-15 cells and hence that H_2_O_2_ triggered apoptosis. 

The mechanism of neuroprotection by mesuagenin C **3** remains to be clarified. Since the release of cytochrome c from mitochondria which is initiated by the Bax translocation to the mitochondrial membrane is directly linked to the destruction of mitochondrial integrity, which in turn triggers caspase activation, thus inhibiting this crucial step should impede neuronal apoptosis thereby rescue dying neurons. The release of cytochrome c from mitochondria to the cytosol is essential for caspase-3 activation and activates downstream cell death pathway [55]. In the current study, we have successfully shown that mesuagenin C **3** suppressed H_2_O_2_-induced activation of caspase-3/7. Because caspase-3 is an effector caspase, whose activation occurs in the execution phase to kill cells indiscriminately, our data suggest that mesuagenin C **3** protects cells by reversing apoptosis. Since the activation of caspase-3/7 is also believed to be vital for commitment to or execution of neuronal apoptosis [56] therefore the suppressive effect of mesuagenin C **3** on H_2_O_2_-induced caspase-3/7 activity further advocates that the protective effect of mesuagenin C **3** on cell death is caspase dependent. In addition, our findings also suggest that mesuagenin C **3** may also act by inhibiting the H_2_O_2_-induced caspase-9 activation, the executioner enzyme implicated in the activation of the apoptotic pathway. 

Our present data led us to speculate that mesuagenin C **3** might exert its effects on caspase-9 activity, firstly, by inhibiting proapoptotic proteins like p53, Bax, and Bad or inducing expression of antiapoptotic family members such as Bcl-2 and Bcl-xL [57]. Alternatively, it is possible that it mimics the antiapoptotic family members, thus reducing heterodimerization of Bcl-2 family members which subsequently inhibits release of cytochrome c which results in reduction of apaf-1 and procaspase-9 formation to activate the caspase-9 activity [58–60]. Based on our findings, the alteration in the ratio of Bax/Bcl-2 protein, together with the attenuation of H_2_O_2_-induced caspase-3 and -9 activation, might be responsible for the concomitant disruption of apoptosis by mesuagenin C **3**. These observations provided further evidence that mesuagenin C **3** protects NG108-15 cells from H_2_O_2_-induced apoptosis by blocking the caspase-dependent intrinsic apoptotic pathway. 

## 5. Conclusion

The present study has successfully isolated and identified mesuagenin C **3** from the hexane fraction of *M. kunstleri* as the most potent neuroprotective compound in the present neuroprotection model. Mesuagenin C **3** significantly mitigated the effects of H_2_O_2_-induced apoptosis on externalization of phosphatidylserine, aggrandization of GSH level, *Δ*ψm** dissipation, regulation of Bcl-2 and Bax proteins in conjunction with the attenuation of activated caspases, suggesting that suppression of mitochondrial-mediated apoptotic pathway could be possible mechanism underlying the neuroprotective effects exerted by mesuagenin C **3** in NG108-15 cells. 

## Figures and Tables

**Table 1:** Comparison of ^1^H and ^13^C NMR of mesuagenin C and compound 3 (*δ* in ppm; 400 MHz in CDCl3).

Position	*δ*_ H_, *J* (Hz) mesuagenin C [8]	*δ*_ H_, *J* (Hz) compound **3**	*δ*_ C_ mesuagenin C [8]	*δ*_ C_ compound **3**
2			158.9	158.9
3	6.00 (1H, *s*)	6.01 (1H, *s*)	112.2	112.2
4			157.2	157.3
4a			100.7	100.7
5-OH	5.98 (1H, *s*, 5-OH)	5.99 (1H, *s*, 5-OH)	154.4	154.4
6			112.6	112.6
7-OH	14.54 (1H, *s*, 7-OH)	14.54 (1H, *s*, 7-OH)	167.1	167.0
8			103.8	103.8
8a			155.8	155.8
1^′^			137.1	137.1
2^′^	7.40 (1H, *m*, Ar)	7.42 (1H, *m*, Ar)	127.6	127.6
3^′^	7.52 (3H, *m*, Ar)	7.54 (3H, *m*, Ar)	129.6	129.5
4^′^			130.2	130.1
5^′^			129.6	129.5
6^′^	7.40 (1H, *m*, Ar)	7.42 (1H, *m*, Ar)	127.6	127.6
1^′′^	3.30 (2H, *d*, *J* = 6.72)	3.31 (2H, *d*, *J* = 6.72)	21.7	21.6
2^′′^	5.08 (1H, *t*, *J* = 7.36)	5.10 (1H, *t*, *J* = 7.36)	120.6	120.6
3^′′^			138.2	138.1
4^′′^	1.68 (3H, *s*)	1.70 (3H, *s*)	16.3	16.3
5^′′^	2.03–1.91 (4H, *m*)	2.04–1.92 (4H, *m*)	39.8	39.8
6^′′^			26.6	26.6
7^′′^	4.99 (1H, *t*, *J* = 7.32)	5.00 (1H, *t*, *J* = 7.32)	124.1	124.0
8^′′^			131.7	131.7
9^′′^	1.53 (3H, *s*)	1.54 (3H, *s*)	17.6	17.7
10^′′^	1.59 (3H, *s*)	1.60 (3H, *s*)	25.8	25.8
1^′′′^			210.7	210.8
2^′′′^	4.11 (1H, *m*)	4.12 (1H, *m*)	40.5	40.5
3^′′′^	1.28 (6H, *d*, *J* = 7.32)	1.29 (6H, *d*, *J* = 7.32)	19.4	19.4
4^′′′^			19.4	19.4

**Table 2:** Neuroprotective effects of 4-phenylcoumarins (200 *μ*M) isolated from hexane bark of *M. kunstleri *against H_2_O_2_-induced apoptosis in NG108-15 cells.

Compound^a^	Cell viability (%)^b^
Control	100
H_2_O_2_^c^	45.41 ± 3.42^#^
1	42.41 ± 2.35*
2	51.46 ± 1.42*
3	78.99 ± 3.49*
4	61.99 ± 1.58*
5	56.47 ± 1.70*
EGCG (50 *μ*M)^d^	85.19 ± 2.17

The values shown are the means ± SE of at least three independent experiments.

^a^NG108-15 cells were pretreated with 4-phenylcoumarins (3.125–200 *μ*M) for 2 h prior to exposure to H_2_O_2_ (2 mM). After incubation, cells were assessed by MTT to determine the percentage viability.

^b^Cell viability was measured by MTT assay.

^c^H_2_O_2_-treated value differed significantly from the untreated control at the level of ^#^ *P* < 0.05.

^d^EGCG was used as standard positive control.

^#^Results differ significantly from H_2_O_2_-treated group compared to the control untreated group: *P* < 0.05.

^∗^Significantly from H_2_O_2_-treated group compared to the treatment group: *P* < 0.05.
